# Treatment of postoperative jejunal intussusception in adult with oral gastrografin after laparoscopic low rectal resection. A case report

**DOI:** 10.1016/j.ijscr.2020.08.005

**Published:** 2020-08-15

**Authors:** Mafalda Romano, Ernesto Tartaglia, Ferdinando Amodio, Angelo Gragnaniello, Sara Bortone, Massimiliano Fabozzi

**Affiliations:** a Department of General and Oncological Surgery, Hospital “A.Tortora”, Pagani, Italy; b Department of Laparoscopic and Robotic General Surgery, Azienda Ospedaliera dei Colli "Monaldi Hospital", Naples, Italy; c Department of Radiology, Hospital “A.Tortora”, Pagani, Italy

**Keywords:** POD, post-operative day, BMI, Body Mass Index, CT, computed tomography, MRI, magnetic resonance imaging, RT+ CT, chemoradiotherapy, US, abdominal ultrasonography, os, oral, Intussusception, Postoperative bowel intussusception, Intestinal obstruction, Gastrografin, Contrast material

## Abstract

**Introduction:**

Postoperative intussusception is a rare cause of intestinal obstruction in adults. Intussusception refers to a condition in which a segment of the intestine invaginates into the lumen of an adjacent segment of the intestine.

**Presentation of case:**

We report a case of postoperative jejunojejunal intussusception in a 42-year-old male following a laparoscopic low anterior resection for rectal cancer. In post-operative day (POD) 2 the patient showed intermittent bowel obstruction and fever. Diagnosis was established with abdominal computed tomography (CT) and enteroclysis, which led to a spontaneous reduction of the invagination.

**Discussion:**

The incidence of intussusception in adults is rare condition. It represents less than 5% of all cases. In adults, transient asymptomatic enteric intussusception often resolves spontaneously without any treatment. When bowel obstruction is complete and persistent, surgery is need. Intussusception as cause of intestinal obstruction should be kept in mind in a post operative patient who develops obstructive symptoms. Early diagnosis makes the difference between surgical and others treatment. The pathogenesis and diagnosis of postoperative intussusception in the adult is discussed in the case report.

**Conclusion:**

The authors presented a rare cause of postoperative small bowel obstruction treated without surgery. The peculiarity of this case report is that the authors have successfully used, in an adult, conservative treatment with gastrografin, which is the treatment of choice used in children. The use of gastrografin was decisive and there were no recurrences in the follow up.

## Highlights

• Intussusception is a rare cause of postoperative small bowel obstruction in adult and the treatment is still debated.
• Bowel Intussusception should be kept in mind in a post operative patient who develops obstructive symptoms.
• The time of diagnosis makes the difference between surgical treatment and others.
• Abdominal CT have a good accuracy in diagnosis.
• Surgery is necessary if peritoneal irritation is present. Otherwise, conservative treatment is considered.

## 1 Introduction

Intestinal intussusception is a relatively common abdominal emergency in children; however, the incidence of intussusception in adults is rare and represents less than 5% of all cases [1]. The pathogenesis of postoperative bowel intussusception is not clear, and clinical examination usually doesn’t reveal a possible cause or pathologic leading point following surgery [3].

Symptoms of adult intussusception usually are nonspecific, such as nausea, vomiting, and abdominal pain. It's difficult to distinguish an intussusception from a paralytic ileus [2].

Bowel surgical resection is the most frequent treatment of the obstruction, but it has to be considered that the intussusception reduction can be obtained with hydrostatic reduction as well, with no anastomosis compromission [4]. In our case report, the patient didn’t show signs of peritoneal irritation, offering a good indication for hydrostatic or pneumatic reduction. This work has been reported in line with the SCARE criteria [5].

## 2 Presentation of case report

A 42 years-old male patient, with a BMI of 19.2, came to our observation for rectal bleeding in May 2019. The patient did not assume any medication, had no allergies and did not smoke. There was no familiarity with rectal cancer. After a clinical visit, he performed a colonoscopy with diagnosis of *neoformation of 4 cm, occupant half of the colic lumen* of the rectum, 4 cm from the anal verge. The biopsies showed *high-grade gastrointestinal mucosal neoplasia.* No secondaryisms was founded. A total body CT and abdomen Magnetic Resonance imaging (MRI) was used for staging.

On July 2019 the patient started neoadjuvant chemoradiotherapy (RT+ CT treatment). After 4 months from the neoadjuvant therapy, the patient was re-evaluated with CT scans abdomen and colonoscopy showing a significant reduction of the tumor. A tatoo of the lesion was performed.

The patient was admitted to our Surgical Department and performed intestinal mechanical preparation before surgery. An ultra-low videolaparoscopic resection of the rectum was performed.

In post-operative day (POD) 1, vital parameters were physiological and urinary catheter was removed. The patient was mobilizes and started the water diet. The abdominal drainage was about 75 cc of serum.

**Fig. 1:**
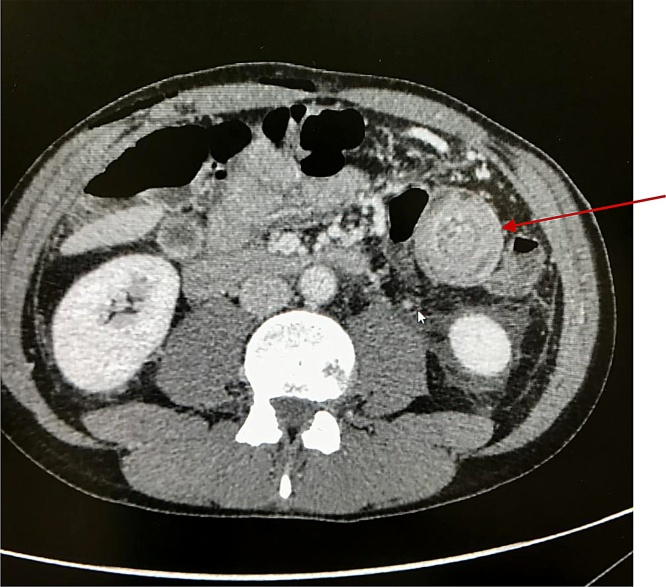
**CT scan image**: The arrow indicates invagination of intestinal loop in the left hypochondrium with endolume corpuscular fluid component.

**Fig. 2:**
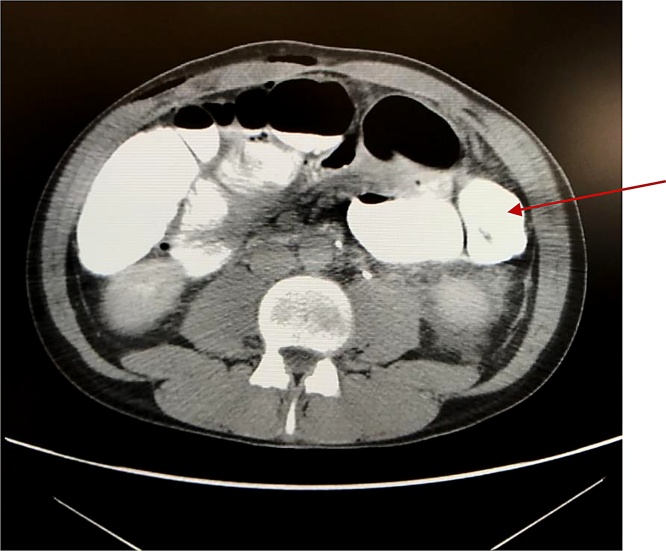
**CT scan image**: The arrow indicates the subsequent check-up performed after administration of gastrografin for os, showing the resolution of the obstruction.

In POD2, the patient made flatus, abdominal drainage was removed and vital parameters were still normal. During the same day the patient showed fever and abdominal pain. Antibiotic therapy was restored. The patient underwent urgent abdomen CT with contrast showing the invagination of intestinal loop (Fig. 1) in the left flank with sign of mechanical occlusion. We administered two glasses of gastrografin diluted in water (about 150 mL for each glass: half glass of water and half glass of gastrografin) for os, with no hydrostatic pressure. Patient had been informed and consent was obtained for the procedure, performed by chief of general surgery with collaboration of a radiologist. After administration of gastrografin for os, the previous signs of obstruction appeared to progressively resolve (Fig. 2).

In POD3 the abdomen and chest CT contrast showed no pathological findings.

In POD4, the patient was fever-free with treatable abdomen and channeled to feces and flatus.

The patient avoided another surgery, solving the problem thanks to a minimally invasive method.

The patient was discharged in POD6 and he was instructed on the type of nutrition to follow at home. In addition, outpatient appointments and cancer visits were scheduled.

## 3 Discussion

Intussusception is similar to an “internal prolapse” of the proximal bowel within the distal bowel [1]. The consequences are bowel obstruction and ischemia, due to compromising of the mesenteric vascular flow. The incidence of intestinal intussusception in children is a common emergency, while in adults is rare [1]. In the latter, asymptomatic bowel intussusception can be resolve spontaneously and it is often noted on occasional radiological imaging with no treatment need [6]. Instead, complete and symptomatic small bowel obstruction due to intussusception is less frequent and can cause bowel perforation which required surgical intervention [1]. Another possible treatment is hydrostatic reduction, but it may compromise bowel anastomosis [7]. The clinical presentation is usually non-specific characterized by abdominal pain, nausea, vomit. The abdominal exam is often non-diagnostic, which contributes to an error or delay in diagnosis [8].

Once bowel intussusception is suspected, CT scan can be helpful to make the differential diagnosis between a complete small bowel obstruction, adhesion-related obstructions and non-adhesions pathology [8,9].

However, considering the episodic nature of intussusception, the CT scan must be performed on the onset of discomfort, and not in between episodes because as spontaneous reduction may occur [9].

The gastrointestinal contrast agent could define the real nature of intussusceptions [10], but it has never been documented as a reliable instrument to reduce intussusception [8], because of possible recurrence of the intussusception.

The characteristics of intussusception in adults on CT scan are an early “Target or sausage-shaped mass” with eccentrically located areas of low density [11], as shown in Fig. 1. A layering effect is shown when the longitudinal compression and venous congestion occurs. The diagnostic accuracy of abdominal CT has been reported to be around 58–100% [12,13].

Intussusception can be diagnosed also using abdominal ultrasonography (US) which may cause severe wound pain, with patient discomfort and image artifacts [9].

There are several hypotheses regarding potential causes including overzealous or impaired peristalsis, prolonged ileus, increased abdominal pressure, fibrous adhesions around the suture site [14].

In adults, the postoperative intussusception can be idiopathic or secondary.

Idiopathic postoperative intussusception is more frequent in adults from 45 to 51 years old, and it develops in POD 4 or 5, and is typically jejunojejunal [15].

Secondary intussusception seems due to a bowel peristalsis change associated to alterations of the bowel wall or irritations of the lumen. This is the trigger mechanism of the bowel invagination [1,16,17].

Reymond et al. [18,20] have 2 hypotheses about post-operative intussusception: in the first case, it may start from a functionally non-contractile part of the intestinal wall; in the second case, the presence of any adhesions between two non adjacent bowel loops, could determine an extra luminal lead point. This lead to kinking point of the bowel to induce an intussusception [19].

Ein et al. [20] suppose that another possible cause of alteration of the peristalsis in postoperative period is the excessive manipulation of the bowel during surgery with consequent lesions of the bowel wall.

The classical clinical features are similar to those of postoperative small bowel obstruction and It is difficult to differentiate postoperative intussusception from paralytic ileus in patients with common clinical symptoms such as nausea, vomiting, abdominal pain and failure to pass a flatus [2]. Indeed, *Hussain et al.* [8] warned of delayed diagnosis of postoperative intussusception. Their case was diagnosed 22 days after surgery because the patient presented with common symptoms. A delayed diagnosis may change the treatment. In our case, the diagnosis was made on POD 2, based on CT scans and this allowed us to do not operate the patient.

However, spontaneous reduction has been reported in some cases [9]. Indeed, the CT scan must be performed on the onset of presentation of clinical symptoms to make the diagnosis.

Surgery is necessary if peritoneal irritation is present in patients with intussusception. In patients with no sign of peritoneal irritation, hydrostatic or pneumatic reduction is indicated, but when it fails or in case of recurrence surgery is needed. However, timing and type of treatment is still subject of debate [21] (Fig. 1).

In our case, the patient was treated with gastrografin-reduction CT-guided. Ianora et al. [10] demonstrated that “hyperdense” contrast agent (gastrografin) could be more effective in short and long term resolution, as we describe in our case. The pharmacological mechanism of the active ingredient of gastrografin, is represented by the creation of an osmotic gradient, linked to the electrolyte dissociation of salt that leads to the increase of the net water content at the intemital obstructal level. The considerable osmotic gradient created by the solution results in progressive dehydration of the intestinal wall. This mechanism causes an increase of intraluminal pressure, intraluminal volume and peristalsis that are able to resolve the invagination.

## 4 Conclusion

Post operative intussusception is a rare occurrence and is difficult to diagnose clinically. Intussusception as a cause of intestinal obstruction should be kept in mind in a post operative patient who develops obstructive symptoms. The time of diagnosis makes the difference between surgical treatment and the possibility to easily administer gastrografin per os for an adequate solution of the obstruction.

## Declaration of Competing Interest

No conflict of interest for all authors.

## Sources of funding

No funding was requested.

## Ethical approval

No specific ethic approval was necessary, because case report is related to an operative procedure necessary for health of patient.

## Consent

Written informed consent was obtained from the patient for publication of this case report and accompanying images. A copy of the written consent is available for review by the Editor-in-Chief of this journal on request.

## Author contribution

Romano Mafalda, Fabozzi Massimiliano: Conceptualization;

Amodio Ferdinando: Data curation;

Tartaglia Ernesto: Formal analysis;

Gragnaniello Angelo: Funding acquisition;

Romano Mafalda, Fabozzi Massimiliano, Tartaglia Ernesto Investigation;

Tartaglia Ernesto: Methodology;

Romano Mafalda: Project administration;

Romano Mafalda, Fabozzi Massimiliano: Resources;

Tartaglia Ernesto: Software;

Romano Mafalda, Fabozzi Massimiliano: Supervision;

Romano Mafalda: Validation;

Bortone Sara, Amodio Ferdinando: Visualization;

Romano Mafalda: Roles/Writing - original draft;

Tartaglia Ernesto, Fabozzi Massimiliano: Writing - review & editing.

## Registration of research studies

N/A.

## Guarantor

A guarantor of data is corresponding author, Dr. Mafalda Romano.

## Provenance and peer review

Not commissioned, externally peer-reviewed.
